# Crosstalk among podocytes, glomerular endothelial cells and mesangial cells in diabetic kidney disease: an updated review

**DOI:** 10.1186/s12964-024-01502-3

**Published:** 2024-02-19

**Authors:** Shiwan Hu, Xing Hang, Yu Wei, Han Wang, Lili Zhang, Linhua Zhao

**Affiliations:** 1 Institute of Metabolic Diseases, Guang’ anmen Hospital, China Academy of Chinese Medical Sciences, Beijing, 100053 China; 2 Beijing University of Chinese Medicine, Beijing, 100029 China

## Abstract

Diabetic kidney disease (DKD) is a long-term and serious complication of diabetes that affects millions of people worldwide. It is characterized by proteinuria, glomerular damage, and renal fibrosis, leading to end-stage renal disease, and the pathogenesis is complex and involves multiple cellular and molecular mechanisms. Among three kinds of intraglomerular cells including podocytes, glomerular endothelial cells (GECs) and mesangial cells (MCs), the alterations in one cell type can produce changes in the others. The cell-to-cell crosstalk plays a crucial role in maintaining the glomerular filtration barrier (GFB) and homeostasis. In this review, we summarized the recent advances in understanding the pathological changes and interactions of these three types of cells in DKD and then focused on the signaling pathways and factors that mediate the crosstalk, such as angiopoietins, vascular endothelial growth factors, transforming growth factor-β, Krüppel-like factors, retinoic acid receptor response protein 1 and exosomes, etc. Furthermore, we also simply introduce the application of the latest technologies in studying cell interactions within glomerular cells and new promising mediators for cell crosstalk in DKD. In conclusion, this review provides a comprehensive and updated overview of the glomerular crosstalk in DKD and highlights its importance for the development of novel intervention approaches.

## Introduction

Diabetes mellitus is a pervasive and complex chronic disease that afflicts over 500 million individuals worldwide. Projections by the International Diabetes Federation suggest that this number will escalate to 7 billion by the year 2045 [1]. As diabetes mellitus advances, it precipitates a myriad of complications, encompassing cardiovascular diseases, diabetic neuropathy, diabetic retinopathy, and diabetic kidney disease (DKD) [2]. DKD constitutes a substantial risk factor for both cardiovascular disease and end-stage renal disease (ESRD) [3], emerging as a prominent contributor to mortality in individuals afflicted with type 1 and type 2 diabetes [4]. Clinically, DKD manifests as proteinuria, podocyte dedifferentiation, epithelial-mesenchymal transition, heightened urinary albumin excretion rate (UAER), and elevated blood pressure [5]. These pathological cascades may extend for a duration exceeding a decade [6], culminating inexorably in ESRD. Several reports indicate that alterations in glomerular structure and function play an important role in DKD [7–9]. Alterations of glomerular involve GECs, podocytes, and MCs. Podocytes and GECs are physically close and separated by the glomerular basal membrane (GBM), together they make up the GFB [10]. The GFB comprises the GBM, fenestrated endothelium and its associated glycocalyx, podocyte foot processes, and the slit membrane (SD). Impairment of any component of the GFB can result in persistent proteinuria. Under physiological conditions, the growth, survival, differentiation and permeability can be regulated by paracrine pathways between podocytes and endothelial cells [11]. MCs are intricately linked to the GFB, and their contractile behavior orchestrates dynamic alterations in the geometrical configuration of glomerular capillaries [12], which together establish a biomechanical unit capable of creating tubular wall tension. This architectural framework facilitates the prerequisites for physiological intercellular communication within the glomerulus. Emerging evidence underscores the pivotal role of cell-cell crosstalk in driving the progression of DKD. This intricate interplay becomes particularly evident during processes such as endothelial dysfunction, mesangial expansion, podocyte loss, and progressive podocyte apoptosis, ultimately culminating in accelerated glomerulosclerosis and functional deterioration [13–15]. Presently, the predominant research focus in DKD appears to center on the intricate interplay among cells, primarily highlighting interactions between GECs and podocytes. However, it is imperative to underscore the pivotal role of MCs in preserving glomerular homeostasis, as emphasized in a recent investigation [16], because MCs assume a critical function not only in orchestrating the normal assembly of glomerular capillary clusters but also in facilitating the production of podocyte-specific vascular endothelial growth factor-A (VEGF-A), fostering the proper maturation of GECs, and governing other pivotal physiological processes [17]. The precise mechanisms governing the cytokine interplay among MCs, GECs, and podocytes, which collectively trilaterally influence cognate receptors on recipient cells, remain incompletely elucidated. An initial summary encompassing a series of remarkable investigations delving into the intricacies of cellular interactions among podocytes, GECs, and MCs in diverse glomerular diseases has been presented [18]. Nonetheless, it may been advocated for further comprehensive exploration within this realm recently.

In this comprehensive review, our attention has been directed toward the pathological transformations occurring in three distinct cell populations: GECs, podocytes, and MCs. Furthermore, we have delineated the intricate web of glomerular crosstalk that underpins the development of DKD. This exposition serves to elucidate the mechanisms governing intraglomerular crosstalk, thereby contributing to an enhanced comprehension of DKD pathogenesis from a cellular and molecular biology perspective. Additionally, we also list current advanced techniques for studying cell-cell interaction in kidney diseases to better understand the mechanisms of cell-cell crosstalk, affording the latest insights for the precise delineation of potential intervention strategies.

## Injury of glomerular cells in DKD

**Fig. 1:**
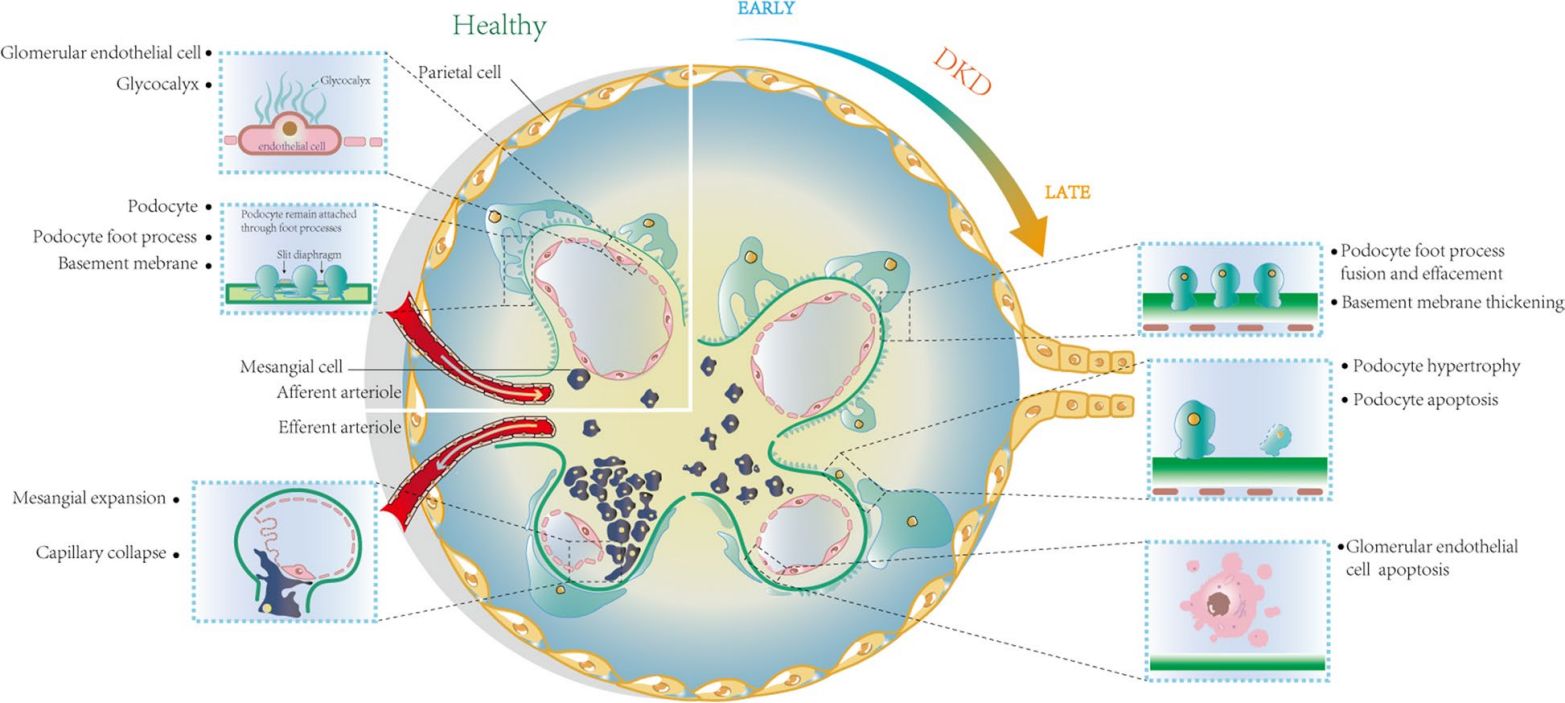
Pathological structural changes of glomerular cells in DKD. Healthy glomeruli includes glomerular endothelial cells, parietal cells, basement membrane, podocytes, podocyte foot process and MCs; The glomerular manifestations of DKD include podocyte foot process effacement, basement membrane thickening, podocyte hypertrophy, podocyte apoptosis, apoptosis of GECs and mesangial expansion

Glomerular cells primarily consist of GECs, podocytes, and MCs. In the context of DKD, these three cell types undergo injury characterized by distinct alterations in cellular morphology (Fig. 1), consequently affecting the fundamental functions of glomerular filtration.

### MCs injury in DKD

MCs represent intrinsic glomerular inhabitants, strategically positioned between glomerular capillary loops and closely juxtaposed to GECs or the GBM. In most instances, MCs and GECs are tightly packed together, separated solely by a narrow extracellular cleft. Occasionally, MC protrusions have been observed to penetrate the intercellular gap between endothelial cells, establishing direct connections with the capillary lumen [12]. MCs wield control over the surface area of glomerular capillaries through their contractile behavior, thereby significantly influencing glomerular filtration rates (GFR) [19]. This process is subject to modulation by alterations in glomerular hemodynamics precipitated by factors such as Angiotensin II [20].

Under various pathological conditions, including high glucose (HG) levels, hyperfiltration, elevated intraglomerular pressure, and advanced glycation end products, mesangial activation transpires, marking one of the initial pathological events in MCs [21]. This phenomenon is recognized as phenotypic transdifferentiation, characterized by the expression of actin and α-smooth muscle actin [22, 23]. Activation of MCs is closely linked to mesangial expansion and the eventual development of glomerulosclerosis. Compared with GBM thickening, mesangial expansion plays a more important role in the reduction of GFR and relates to proteinuria [24]. In the context of DKD, the initial alteration within the glomerulus is the thickening of the GBM, followed by mesangial expansion which encompasses the abnormal proliferation of MCs and pathological accumulation of matrix proteins within the central glomerular region [20, 25, 26]. As mesangium expands, it comes into contact with the innermost regions of the thickened GBM, leading to the detachment of capillaries from the GBM and initiating their collapse [27]. Mesangial expansion is widely recognized as an indicator of the progression of diabetic nephropathy from its early or moderate stage to a more advanced stage [28, 29], culminating ultimately in advanced diabetic glomerulosclerosis [27]. A hallmark of glomerulosclerosis is the obstruction of glomerular capillaries by extracellular matrix (ECM) [30]. The renin-angiotensin-aldosterone-system (RAAS) assumes a pivotal role in this process, with angiotensin II (AngII) playing a central role in DKD [31]. AngII has been implicated in promoting glomerular mesangial expansion, GBM fragmentation, increased ECM deposition, and consequent collagen synthesis, thereby exacerbating renal injury [32, 33]. Moreover, it has been reported that AngII contracts MCs by activating the angiotensin II type 1 receptor (AT1 receptor), representing a crucial factor in glomerulosclerosis [34]. Additionally, aldosterone is believed to upregulate plasminogen activator inhibitor 1, promote macrophage infiltration, mediate the proliferation of MCs and ECM, and contribute to renal fibrosis [35]. Under pathological conditions, the ability of MCs to regenerate, proliferate, and produce ECM further exacerbates glomerulosclerosis [36]. In addition to this, tumor necrosis factor-α (TNF-α) and connective tissue growth factor (CTGF) have been implicated in several pro-sclerotic pathways that contribute to the progression of mesangial expansion and DKD [37, 38]. Recent studies have also shed light on the role of RNA within MCs, with evidence suggesting that miR-422a and miR-15b-5p in DKD not only drive increased matrix production by MCs but also lead to MC apoptosis [39, 40]. MCs, along with their associated matrix, constitute the central stalk of the glomerulus, functioning as a part of an integrated unit in close communication with endothelial cells and podocytes [41]. Considering the frequent observation that podocyte injury often results in MC proliferation, and vice versa, where MC injury leads to foot process fusion and proteinuria, it is highly likely that cytokine crosstalk is a prevalent phenomenon within this dynamic interplay.

### GECs injury in DKD

GECs represent a pivotal component of the GBM. These cells envelop the luminal surface of glomerular capillaries, coming into direct contact with circulating blood. GECs are characterized by fenestrations, with fenestrae measuring approximately 70-90 nm [42]. This architectural feature assumes a pivotal role in glomerular filtration and urine formation, thereby regulating glomerular filtration function. The apical surface of GECs is ensconced within a negatively charged endothelial glycocalyx and an endothelial surface layer (ESL) [43], which play integral roles in upholding the integrity of the GFB.

GECs are particularly susceptible to the influence of blood glucose levels, rendering them prone to damage or dysfunction. Increased oxidative stress, pro-inflammatory activation, and perturbations in cell signaling have all been documented contributors to the progression of endothelial dysfunction observed in DKD [44]. Within the milieu of DKD, signaling pathways governing endothelial nitric oxide synthase (eNOS) activation in GECs undergo alterations, leading to diminished nitric oxide (NO) production [45] and subsequent GEC injury [46, 47]. Enhanced oxidative stress emerges as a plausible mechanism underpinning GEC injury in DKD [48]. The localized accumulation of excess reactive oxygen species (ROS) within glomerular compartments contributes to glomerular damage, encompassing GEC apoptosis and the attenuation of glomerular glycocalyx expression [49, 50], ultimately culminating in albuminuria [16].

In recent years, several studies have directed their focus towards exploring the phenomenon of endothelial-mesenchymal transition (EndMT) in GECs. Notably, under the influence of HG conditions, the process of EndMT in GECs predominantly involves responses mediated by transforming growth factor-beta (TGF-β) signaling pathways [51]. The consequence of EndMT in GECs includes the development of albuminuria and fibrosis within the glomeruli, resulting in the disruption of the normal structural and functional integrity of the kidney, ultimately leading to ESRD [52, 53]. In summary, the multifaceted injury observed in GECs in DKD is brought about through various mechanisms, with apoptosis being a predominant factor. Recognizing the significance of protecting GECs is crucial for retarding the progression of DKD.

### Podocytes injury in DKD

Podocytes, being terminally differentiated specialized pericyte-like cells, are adhered to the exterior of the GBM where they collaboratively form the GFB alongside GECs [54]. Podocytes extend numerous foot processes from their cell bodies, which attach to the GBM and collectively form the slit diaphragms. These slit diaphragms serve as the ultimate barrier preventing the passage of proteins into the urinary filtrate, and the contraction and expansion of podocyte foot processes regulate the filtration function of the glomerulus [55]. In HG conditions, the podocyte’s morphological changes will occur, which means the injury of the podocytes. The main morphological and functional changes of podocytes in DKD involve hypertrophy, foot process effacement, epithelial-mesenchymal transdifferentiation (EMT), apoptosis and autophagy [56].

Oxidative stress has been established as the underlying cause of podocyte hypertrophy in DKD [57]. Furthermore, increased expressions of factors such as TGF-β1, Angiotensin II (AngII), and mammalian target of rapamycin complex 1 (mTORC1) have been implicated in promoting podocyte hypertrophy in response to HG [58, 59]. Research has identified podocyte EMT as a potential pathway leading to proteinuria [60], with pathways like the Wnt/β-catenin signaling pathway, SDF-1α, and PI3K/AKT signaling pathway being confirmed as promoters of podocyte EMT [61–63]. Podocyte apoptosis can lead to proteinuria and glomerulosclerosis in DKD [64], with two pathways, namely, the extrinsic pathway (centered on extracellular ligands such as tumor necrosis factor - TNF) and the intrinsic pathway (centered on mitochondria-mediated mechanisms) identified as contributors to podocyte apoptosis [65]. Podocyte autophagy, a type II programmed cell death, plays a critical role in the pathogenesis of podocyte loss, leading to extensive proteinuria in DKD [66]. Notably, while podocyte autophagy serves a renoprotective role in early-stage DKD, dysregulation of autophagy occurs in advanced stages, contributing to podocyte injury [67].

## The pathological crosstalk among podocytes, GECs, and MCs in DKD

**Fig. 2:**
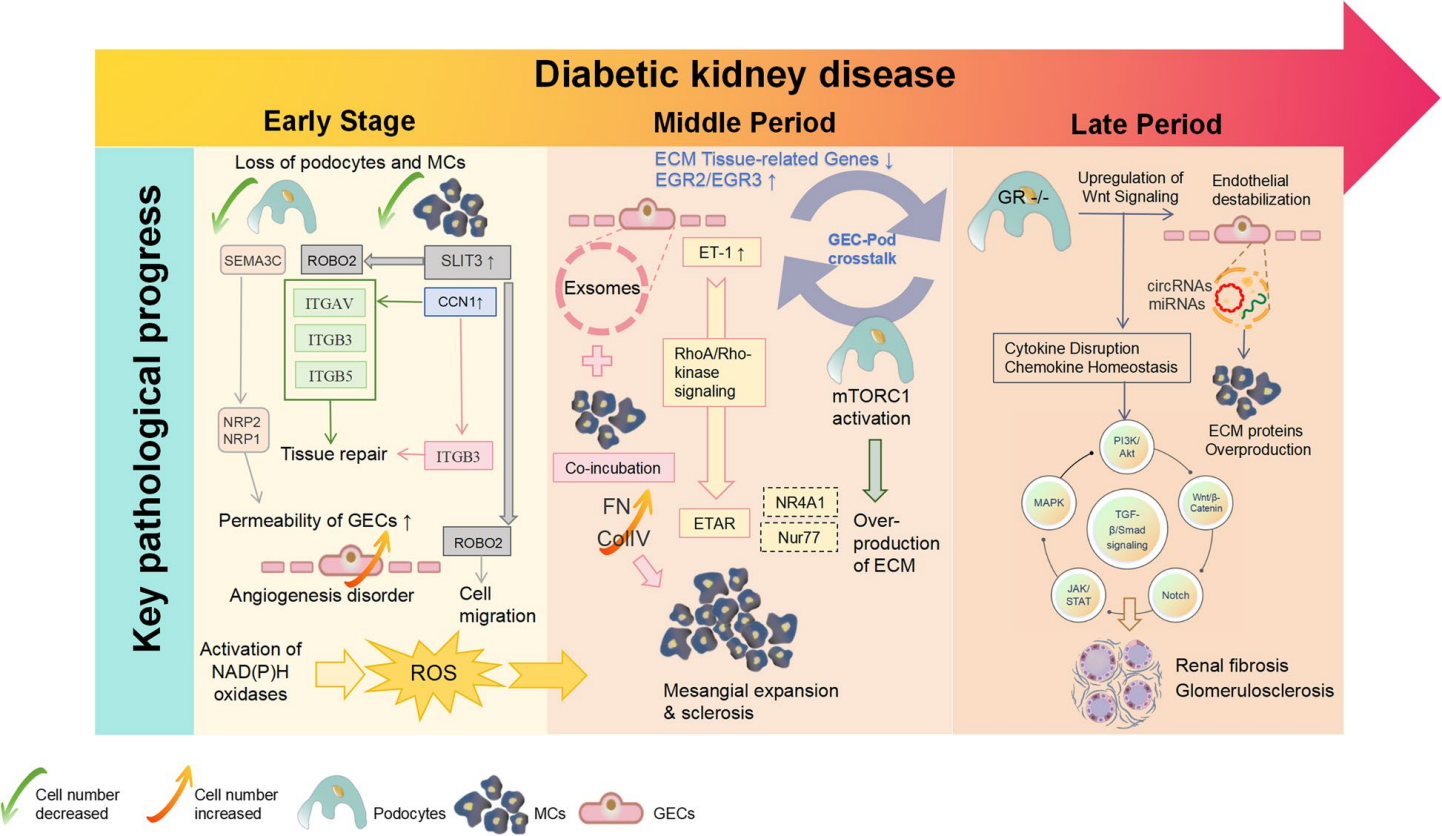
The available evidence to summarize the early to late periods of intracellular cell interactions in the glomerulus. During the progression of DKD, the mechanisms of intracellular cell interactions in the glomerulus at different stages have different emphases. In the early stage of DKD, there is a tendency towards loss of podocytes and angiogenesis disorders, and the glomerular filtration barrier begins to be damaged. With more ROS being produced, the progression of DKD develops further. In the transitional period, mesangial expansion and sclerosis accompanied by overproduction of ECM, gradually lead to renal fibrosis and ultimately develop into the late-stage of DKD. The over-activation of a series of renal fibrotic pathways with TGFΒ as the core and the release of exosomes of related signal transmitters eventually aggravate the renal fibrotic process of DKD, resulting in a sharp decline in renal function and developing into the end-stage renal disease

The glomerulus constitutes the primary functional unit of renal filtration, with MCs serving to maintain renal structural integrity. The filtration barrier includes GECs, podocytes, and the GBM [68]. Previous studies have unveiled potential roles for interactions between these three cell types during the progression of DKD [15, 69, 70]. The following section provides an overview of the latest available evidence concerning the distinct phases of intracellular interactions within the glomerulus (Fig. 2) and the underlying mechanisms and pathways governing these cellular interactions (Table 1).

**Table 1:** The summary of cellular crosstalk among glomerular endothelial cell, podocyte, and mesangial cells in DKD

Cell Types in the Crosstalk	Mediators	Related Pathways or Mechanisms	Physiological/Pathogenic effects on DKD	Reference(s)
GECs → MCs	Exosome containing TGF-β1 mRNA	TGF-β/Smads signaling pathways	Causing significant mesangial expansion, proliferation and ECM protein overproduction	[71, 72]
GECs → MCs	Exosomes enriched in circRNAs	PI3K/AKT signaling pathway, MAPK signaling pathway	Promoting α-SMA expression and inducing EMT in MCs	[72]
MCs → GECs	Integrin αvβ8 and its main ligand TGF-β	TGF-β/Smads signaling pathway (probably)	Reducing TGF-β binding; Causing bioactive TGF-β release, thus stimulating apoptosis of GECs	[73]
GECs → Podocytes	Exosome containing TGF-β1 mRNA	Wnt/β-catenin signaling pathway	Causing EMT and dysfunction of podocytes	[74]
GECs → Podocytes	Bone morphogenetic protein and activin membrane-bound inhibitor	TGF-β/ALK1-Smad1/5 signaling pathway	Worsening podocyte loss; Inducing proteinuria	[75]
MCs → Podocytes	Exosome containing TGF-β1 mRNA	TGF-β1/PI3K/AKT signaling pathway	Inducing podocytes’ apoptosis and inhibiting cell adhesion	[76]
Podocytes → GECs	Ang-1 expressed in podocytes; Tie-2 expressed in GECs	Ang-1/Tie-2 signaling pathway	Promoting the survival of GECs; Decreasing proteinuria; Preventing abnormal proliferation, angiogenesis, and migration of GECs	[77–79]
Podocytes → GECs	Ang-1 expression in podocytes; Tie-2 expressed in GECs	Ang-1/Tie-2 signaling pathway	Modulating podocyte injury responses and secretion of key angiogenic factors, thereby affecting GECs remodeling after injury	[79]
Podocytes → GECs	Ang-2 secreted by podocytes; Tie-2 expressed in GECs	Ang-2/Tie-2 signaling pathway	Leading to more proteinuria and apoptosis of GECs; Inhibiting the maintenance of the integrity of GECs and filtration barrier function	[80]
ECs → MCs	Ang-2 increased and released from ECs under an HG condition;	Ang-2/Tie-2 signaling pathway; miR-33-5p-SOCS5 loop	Inducing MC apoptosis	[81]
Podocytes → GECs	VEGF-A produced by podocytes; VEGF-A receptors on GECs named VEGFR-1 and VEGFR-2	VEGF signaling pathways	Maintaining endothelial fenestration, thereby preserving endothelial function	[82]
Podocytes → GECs	The HG condition up-regulated the production of VEGF-A in podocytes	VEGF signaling pathways	Leading to endothelial cell damage	[83]
GECs → Podocytes	GEC-derived excessive miR-200c; VEGF-A in podocytes	VEGF-A/VEGFR2 signaling pathways	Impairing glomerular homeostasis; Leading to the damage of the glomerular; Causing extensive foot process effacement and proteinuria	[84]
Podocytes → GECs	Beclin-1 in podocytes; VEGF-A secreted by podocytes	VEGF-A/VEGFR2 signaling pathways	Being indispensable for VEGF secretion; Maintaining GFB function; Deleting Beclin-1 in podocytes led to early-onset glomerulosclerosis	[85]
Podocytes → GECs	Overexpressed Sema3A in mature podocytes	Sema3A/NRP1 signaling pathway; VEGF-A/NRP1 signaling pathways	Causing glomerular hypoplasia and apoptosis of GECs	[86]
Podocytes → GECs	VEGF-C over-expressed in podocytes; VEGF receptors	VEGF-C/VEGFR signaling pathways	Reducing the loss of GECs fenestrations	[87]
Podocytes → MCs	Podocyte-specific over-expressed VEGF	PDGF-B-mediated signaling pathways	Decreasing MC markers such as α-SMA, desmin, and PDGFR-β significantly	[88]
Podocytes → MCs	VEGF-A over-expressed in podocytes	VEGF-A/VEGFR signaling pathways	Inducing mesangial expansion	[89]
Podocytes → MCs	VEGF-A overexpressed in podocytes	VEGF-A/VEGFR signaling pathways	Maintaining the survival and differentiation of MCs	[90]
Podocytes → GECs	Edn1 secreted by podocytes	Edn1/ Ednra signaling pathways	Destroying endothelial cells by activating oxidative stress	[91]
Podocytes → GECs	Increasing circulating Edn1	Edn1/ Ednra signaling pathways	Inducing endothelial oxidative stress; Ameliorating mitochondrial ROS in GECs by HG, podocyte loss, albuminuria and glomerulosclerosis	[92]
Podocytes → GECs	Edn1 derived from podocytes; SUMO-specific peptidase 6	Edn1-mediated crosstalk between podocytes and GECs	Exacerbating podocyte loss and GECs dysfunction by HG	[13]
Podocytes → MCs	Ednra and Ednrb specifically deleted in podocytes	β-catenin and NF-κB signaling pathways mediated by endothelin receptors	Avoiding the podocyte loss; Inhibiting the mesangial expansion	[93]
MCs → podocytes	ERAD-related proteins such as phosphorylated IRE1α, Derlin-1, and Derlin-2	ERAD-related signaling pathway	Causing the aggravation of albuminuria and more podocytes’ apoptosis	[93]
GECs → Podocytes	Pro-apoptotic paracrine signaling factors	Mitochondrial dysfunction and oxidative stress	Increasing podocyte apoptosis, cell shrinkage, and some detachment, the increasing level of caspase 3 and cytosckeleton rearrangement	[94]
GECs → MCs	GECs derived NO	Nitric oxide-mediated signaling pathways	Inducing cGMP formation in MCs in a NO dependent manner, related to the regulation of the intraglomerular capillary flow	[95]
GECs → Podocytes	eNOS derived NO	Nitric oxide-mediated signaling pathways	Supporting podocytes with eNOS derived NO to maintain their structure and function, and loss of GECs provokes NO deficiency that precedes podocyte injury	[96]
GECs → MCs	eNOS which is deficient in diabetes mice	Nitric oxide-mediated signaling pathways	Appearing mesangial lysis and late mesangial dilatation, form nodular or Kimmelstiel-Wilson like lesions which means the development of DKD	[47, 97]
GECs → Podocytes	NO derived from GECs	Nitric oxide-mediated signaling pathways	Maintaining podocyte structure and function	[98]
Podocytes → GECs	HIF-1α and SENP1 in podocytes under the hypoxia condition	HIF-related signaling pathways; VEGF/VEGFR2 signaling	Promoting HIF-1α stabilization and activation by increasing SENP1 expression in podocytes, thereby maintaining the survival of GECs and angiogenesis	[99]
GECs → Podocytes	Heterozygous knockout of KLF2 in endothelial cells	KLF2-related signaling pathways	Causing more proteinuria, more podocyte damage, and elevating the expression of angiogenesis markers	[100]
GECs → Podocytes	Heterozygous knockout of KLF2 in endothelial cells	KLF2-related signaling pathways	Reducing the number of podocytes and the expression of podocyte markers	[101]
GECs → Podocytes	Chronic LSS-dependent mediators released from GECs	ERK5-related pathway	Improving the anticoagulants and anti-inflammatory phenotypes, and directly affecting podocyte function in co-culture	[102]
GECs → Podocytes	RARRES1 overexpression in endothelial cells; receptor tyrosine kinase Axl	NK-κB signaling pathway	Inducing podocyte injury	[103]
GECs → MCs	PDGF-B localizes to the GECs, and PDGFR-β localizes to the MCs	PDGF-B/PDGFR-β signaling pathways	Recruiting MCs into developing glomeruli and promoting the formation of capillary rings	[104, 105]
GECs → MCs	Both up-regulated PDGF-B and PDGFR-β in the histologically early stage of DKD	PDGF-B/PDGFR-β signaling pathways	Promoting DKD by releasing paracrine signaling mediators to cause MCs’ damage	[106, 107]
GECs → MCs	Increasing PDGF-B/PDGFR-β expression under the HG and hypoxia condition	PDGF-B/PDGFR-β signaling pathways	Leading to MCs’ proliferation and mesangial expansion	[108, 109]
	Slit2; Transmembrane Roundabout receptor	Slit2-Robo signaling pathways	Regulating axon guidance, ureteric bud branching, and angiogenesis during kidney development, glomerular filtration	[110, 111]
	Slit2 and VEGF derived from MCs; Slit2 and Robo1 in GECs	Robo1/PI3K/Akt/VEGF signaling pathways	Participating in GECs proliferation, migration, and tube formation; Treating abnormal angiogenesis in early DKD through promoting glomerular vascularization	[112]
Podocytes → MCs	BMP4 specifically expressed in podocytes; Smad1 in MCs	BMP4-Smad1 signaling pathways	Regulating the mesangial expansion in DKD, podocyte loss	[113–115]

### Cell-to-cell crosstalk among Podocytes, GECs, and MCs in different development stages of DKD

DKD is characterized by a complex interplay of various pathophysiological pathways, which can be categorized as metabolic, hemodynamic, and inflammatory [116]. These pathways, driven by hyperglycemia, lead to pathological alterations in the glomerulus, especially within podocytes, and the tubulointerstitium, culminating in increased glomerular albumin permeability (albuminuria) and a subsequent decline in estimated glomerular filtration rate (eGFR). According to international consensus meetings [117], the histological progression of DKD can be divided into four stages: thickening of the GBM as the earliest change, followed by mesangial expansion, mesangial sclerosis characterized by nodular glomerulosclerosis or Kimmelstiel-Wilson nodules, and ultimately, glomerulosclerosis leading to ESKD. Based on the distinct pathological processes occurring at different stages of DKD, the mechanisms underlying the interactions among these three cell types within the glomerulus can be summarized as follows.

#### Cellular crosstalk in early-stage DKD

The kidney is a highly intricate organ, and the pathogenesis of kidney tissue involves complex intercellular interactions within a heterogeneous renal environment. Podocytes, as a critical component of the GFB, undergo damage and loss as early events in the development of DKD [118]. In the early pathological state of DKD, there is an observed increase in the number of GECs alongside a decrease in the numbers of podocytes and mesangial cells, as revealed by single-cell RNA sequencing (scRNA-seq) technology [119]. This observation aligns with the characteristics of vascular endothelial podocyte loss and angiogenesis disruption seen in the early stages of diabetic nephropathy [120]. Recent research has highlighted the role of mesangial cells in mediating crosstalk within the glomerular microenvironment during early DKD via the secretion of semaphorin 3C (SEMA3C) [121]. HG conditions promote SEMA3C secretion by mesangial cells, which, in turn, induces endocytosis of microtubules (MT) and enhances glomerular endothelial cell permeability through pathways involving neurociliary protein 1 (NRP1) and neurociliary protein 2 (NRP2) [121]. This suggests that SEMA3C-mediated communication between mesangial cells and glomerular endothelial cells may contribute to early GEC damage in DKD. Furthermore, inhibition of SEMA3C has been shown to ameliorate HG-induced GEC damage [121]. Additionally, studies have identified upregulation of CCN1 and SLIT3 in mesangial cells in early diabetic states, with CCN1 playing a role in tissue repair through interactions with extracellular proteins expressed in podocytes and endothelial cells, while SLIT3 regulates cell migration via interactions with ROBO2 expressed in podocytes and GECs [122]. Furthermore, it was observed that nicotinamide phosphoribosyltransferase (NAMPT), a regulator of insulin secretion in islet β cells, was expressed in MCs. Conversely, the expression of Insulin Receptor (INSR) was reduced in diabetic podocytes. Additionally, in diabetic GECs, there was an increased expression of latent TGF-β binding protein 1 (LTBP1), which plays a role in regulating the targeting properties of TGF-β complexes [122]. Intrarenal oxidative stress plays a pivotal role in both the initiation and progression of DKD. During the early stages of DKD, HG conditions induce the activation of glomerular endothelial NAD(P)H oxidases, leading to the generation of ROS in GECs. This phenomenon subsequently triggers podocyte activation and mesangial expansion, ultimately exacerbating DKD, which is characterized by proteinuria [50].

Mesangial expansion is a significant structural alteration during the progression of diabetic nephropathy. It typically occurs during the intermediate stages of DKD progression. When mesangial expansion surpasses the regulatory capacity of the glomerulus, it extends into the capillary space. This results in a reduction in the filtration area and a decrease in the filtration rate [24]. Some evidence suggests that during this process, the three types of cells interact with each other. For instance, the co-incubation of MCs with exosomes derived from HG-treated GECs has been shown to upregulate the concentrations of extracellular matrix components like type IV collagen (ColIV) and fibronectin (FN). These components are responsible for excessive extracellular matrix (ECM) production and contribute to mesangial expansion. Furthermore, this co-incubation leads to increased proliferation of MCs [71, 72]^.^

The mechanistic target of rapamycin complex 1 (mTORC1) is a multi-subunit protein kinase complex. Hyperactivation of mTORC1 is closely associated with the injury of intraglomerular cells and is a critical factor in the progression of diabetic complications, including DKD [123, 124]. Activation of mTORC1 in podocytes has been linked to mesangial expansion, which represents a crucial step in the development of DKD [125]. Endothelin 1 (Edn1) is an endothelial cell-derived vasoconstrictor peptide with multifunctional properties. It functions as a vasoconstrictor and a growth factor for MCs and is associated with the progression of kidney diseases. Studies have demonstrated that GECs lacking the Edn1 receptor B (Ednrb) promoted mesangial expansion in a HG environment. Further research has revealed that these effects were modulated by the RhoA/Rho-kinase (ROCK) pathway in MCs through endothelin signaling. Edn1 receptor B (ETBR) is primarily distributed in vascular and glomerular endothelial cells, while Edn1 receptor A (ETAR) is mainly distributed in smooth muscle cells and MCs [126]. It has been reported that MCs and GECs interact in the kidney [107]. A study found that MCs could modulate the ability of GECs to synthesize endothelin-1 (ET-1). Under HG conditions, GECs result in an upregulation of ET-1 secretion, which can bind to ETAR in MCs. This interaction accelerates mesangial proliferation and ECM accumulation through the RhoA/ROCK pathway [127].

Another study established a co-culture system of GECs and podocytes for gene expression analysis and found that both HG and methylglyoxal (MGO) led to the downregulation of genes related to ECM tissue in podocytes. Importantly, this downregulation depended on the cell-to-cell crosstalk between GECs and podocytes [128]. Notably, none of the identified genes were differentially expressed in single cultures of their respective cells, underscoring the critical role of cellular interactions between GECs and podocytes in the early development of DKD [128].

While the role of early growth response-1 (EGR1) in MCs has been extensively studied [129], little is known about the potential roles of EGR family members in GECs or podocytes. However, recent research indicates that under high glucose conditions, not only EGR1, which has been linked to DKD pathogenesis [130], but also EGR2 and/or EGR3 were among the upregulated genes in both GECs and podocytes [128]. Furthermore, another immediate early transcription factor significantly upregulated by HG in GECs is nuclear receptor subfamily 4, group A member 1 (NR4A1). This factor was activated by HG in a mouse DKD model associated with renal insufficiency. However, the specific cell type responsible for upregulating NR4A1 in response to hyperglycemic stimuli has not yet been determined [128]. One of the subgroups of NR4A1, known as Neuron-Derived Clone 77 (Nur77), was found to be increasingly expressed in MCs under HG conditions, warranting further investigation [131].

#### Cellular crosstalk in late-stage DKD

Renal fibrosis is a prominent pathological feature in the late stages of diabetic nephropathy, significantly increasing the mortality rate of patients with advanced diabetes. During the development of renal fibrosis in the late stage of diabetic nephropathy, various signaling pathways, including TGF-β/Smad, MAPK, Wnt/β-Catenin, PI3K/Akt, JAK/STAT, and Notch, interact with each other in complex ways. TGF-β, in particular, interacts with almost all fibrosis-related pathways, making TGF-β signaling pathways dominant in the intricate crosstalk during fibrosis progression [132]. There is substantial evidence of cellular interactions mediated by TGF-β signaling pathways among GECs, podocytes, and MCs, which will be discussed in the subsequent section.

The glucocorticoid receptor (GR) is a nuclear hormone receptor that is ubiquitously expressed in most cell types. It has been identified as a key regulator in the process of renal fibrosis [133]. Exosomes, considered a form of cell-to-cell communication, also play a role in kidney fibrosis. Researchers have discovered that exosomes secreted by HG-induced GECs deliver circular RNAs (circRNAs) and microRNAs (miRNAs) to MCs. This, in turn, leads to the activation, proliferation, and excessive production of ECM proteins in MCs, promoting renal fibrosis [71, 72]. Loss of GR upregulates typical Wnt signaling, a significant contributor to renal fibrosis [134], leading to the disruption of cytokine and chemokine homeostasis followed by endothelial destabilization which exacerbates renal fibrosis in DKD [133]. Additionally, GR has the potential to influence the crosstalk between podocytes and endothelial cells. The absence of GR in podocytes leads to abnormal activation of the Wnt signaling pathway and disrupts fatty acid metabolism. These alterations collectively affect the homeostasis of GECs and contribute to the pathogenesis of DKD [135].

A study that explored cell-to-cell interactions and signaling networks within different kidney cell subsets, using ligand-receptor analysis, revealed stronger interactions between fibroblasts from individuals with DKD and various cell types such as proximal tubule cells, podocytes, and endothelial cells, in comparison to nondiabetic controls and individuals with diabetes but no kidney disease (DM samples) [136]. Importantly, these diverse cell interactions were primarily related to chemokines and were predominantly concentrated in fibroblasts, highlighting the close correlation between renal fibrosis and immune responses in the context of DKD [136].

### Cell-to-cell crosstalk mediated by different signaling pathways and mechanisms

#### Transforming growth factor-β (TGF-β) signaling pathways

Circulating proteins associated with the TGF-β signaling pathway play a crucial role in the crosstalk among GECs, podocytes, and MCs, accelerating the development of DKD. Extracellular vesicles (EVs), specifically exosomes, with a size range of 40 to 160 nm (average ~ 100 nm) in diameter and an endosomal origin, are released by all cells [137]. In chronic glomerular diseases like DKD, the paracrine function of exosomes can be altered, and under HG conditions, the overproduction of exosomes plays a significant role in mediating cellular crosstalk.

Compared to GECs treated with normal glucose (NG), HG-treated GECs secrete more glomerular endothelial exosomes containing TGF-β1 mRNA. These exosomes are taken up by MCs and mediate MC activation through the TGF-β1/Smads signaling pathway. This activation results in significant mesangial expansion, proliferation, and overproduction of ECM proteins [71, 72]. Furthermore, when GECs were incubated with TGF-β1 small interfering RNA (siRNA) under HG conditions, the expression of TGF-β1 mRNA significantly decreased in both GECs and the extracted exosomes. This supports the functional importance of transferred TGF-β1 mRNA by exosomes in the crosstalk between GECs and MCs [71]. Another study revealed that exosomes enriched in circular RNAs (circRNAs) released by HG-treated GECs promoted the expression of α-smooth muscle actin (α-SMA) and induced EMT in MCs. The functions of these differentially expressed circRNAs (DECs) were closely related to the PI3K/AKT and MAPK pathways [72]. Recently, researchers have also shown interest in a novel form of cell-cell communication mediated by exosomes. For instance, exosomes from HG-treated macrophages were found to activate MCs via the TGF-β1/Smad3 pathway both in vivo and in vitro [138].

Integrin αvβ8, with TGF-β as its primary ligand, is involved in cellular interactions between MCs and GECs. This integrin plays a central role in connecting extracellular ligands with the intracellular cytoskeleton, facilitating bidirectional signal transmission across the cell membrane [139]. Integrin αvβ8 is abundantly expressed in the kidney and has been found to be protective for the glomerular structure and function during the development of DKD. Experimental models using streptozotocin (STZ)-induced diabetes have shown a reduction in glomerular integrin expression and deposition [140]. Notably, the absence of Integrin αvβ8 in MCs reduces TGF-β binding, leading to the release of bioactive TGF-β. This, in turn, stimulates apoptosis of GECs, and preliminary investigations suggest that this process may involve the TGF-β/Smads signaling pathway. This result provides evidence for crosstalk between MCs and GECs, highlighting that MC-derived Integrin αvβ8 serves as a protective factor for maintaining GEC integrity [73].

GECs also interact with podocytes through the release of exosomes containing TGF-β1 mRNA. Experiments have demonstrated that HG-treated GECs secrete exosomes rich in TGF-β1 mRNA. These exosomes activate the Wnt/β-catenin signaling pathway, leading to EMT and dysfunction of podocytes [74]. Conversely, exosomes derived from GECs also promote pathological processes in podocytes.

To characterize the molecular changes of GECs that occur in response to podocyte loss, researchers used diphtheria toxin (DT) to artificially cause the depletion of podocytes, and transcriptomic analysis of isolated GECs provided the following overall transcriptomic characteristics of GECs in renal diseases in vivo [141]. The results revealed that the significant molecular changes that occur in GECs are secondary consequences of podocyte loss, and TGF-β1 is one of the key mediators of molecular changes in GECs in diseased mice. This elegant work also confirms many differential expression genes (DEG) in GECs, as well as several potential pathways, in response to the alterations of podocytes. A study found that the elimination of bone morphogenetic protein and activin membrane-bound inhibitor (BAMBI) in podocytes and GECs promote the progress of DKD, which is because BAMBI can negatively regulate TGF-β signaling. Researchers further specifically knocked out the BAMBI gene in GECs(EC-BAMBI^−/−^) and in podocytes (Pod-BAMBI^−/−^) and found the TGF-β/Alk5-Smad2/3 pathway and TGF-β/Alk1-Smad1/5 pathway are respectively activated in GECs and podocytes, which accelerates injuries of these cells. Therefore, we can guess that BAMBI in GECs may exert a potential protective effect on podocytes.

To understand the molecular changes in GECs in response to podocyte loss, researchers employed diphtheria toxin (DT) to induce podocyte depletion artificially. Transcriptomic analysis of isolated GECs revealed the significant molecular changes that occur in GECs are secondary consequences of podocyte loss, with TGF-β1 identified as a key mediator of these changes in diseased mice [141]. This work also confirmed several differentially expressed genes (DEGs) in GECs and highlighted potential pathways influenced by podocyte alterations. Knocking out the bone morphogenetic protein and activin membrane-bound inhibitor (BAMBI) in podocytes and GECs was found to promote the progression of DKD, as BAMBI negatively regulates TGF-β signaling [75]. Further studies revealed that the TGF-β/Alk5-Smad2/3 and TGF-β/Alk1-Smad1/5 pathways were respectively activated in GECs and podocytes in response to BAMBI knockout [75]. However, the podocyte injury and loss in GEC-BAMBI^−/−^ diabetic mice were similar to those in Pod-BAMBI^−/−^ diabetic mice, while the proliferation of GECs was only observed in GEC-BAMBI^−/−^ diabetic mice, indicating the complex crosstalk between GECs and podocytes occurs via TGF-β/Smads signaling pathways [75].

Additionally, exosomes released from MCs induced by HG conditions also contribute to podocyte injury. Recent research has shown that, under HG conditions, exosomes derived from MCs secrete more TGF-β1 and significantly up-regulate the expression of TGF-β1 receptor in podocytes. This leads to podocyte apoptosis and inhibits cell adhesion through the TGFβ1-PI3K/AKT signaling pathway [76].

#### Angiopoietin (Ang)/Tie signaling pathways

Angiopoietin (Ang) is a group of vascular growth factors that play a central role in vascular diseases by regulating endothelial permeability and angiogenesis. The Ang/Tie signaling pathway is associated with inflammation and abnormal angiogenesis and contributes to renal dysfunction in Diabetic Kidney Disease (DKD).

Among the major subtypes of Ang, Ang-1 and Ang-2 are the most extensively studied. In the glomerulus, Ang-1 is primarily expressed in podocytes and serves to limit endothelial permeability by binding to the tyrosine protein kinase receptor 2 (Tie-2) on the surface of endothelial cells. This interaction promotes the survival of GECs [77]. Ang-2, on the other hand, is activated by integrins and competitively inhibits Ang-1 by binding to Tie-2 [142]. In DKD, podocyte-specific overexpression of Ang-1 has been shown to significantly reduce proteinuria and prevent the proliferation of GECs by increasing Tie-2 phosphorylation in adult diabetic mice, indicating a protective role for Ang-1 [78].

Previous research has demonstrated that Ang secreted by podocytes plays a crucial role in mediating angiogenesis, proliferation, and migration of GECs [79]. Inhibiting Ang-1 can modulate podocyte injury responses and the secretion of key angiogenic factors, affecting GEC remodeling after injury [79]. In contrast, Ang-2 is downregulated in normal mature glomeruli but upregulated in DKD and glomerulonephritis [77, 143]. Recent studies have shown that Ang-2 can directly stimulate Tie-2 and lead to pro-inflammatory effects. Hypoxia, inflammation, and high glucose levels can trigger the release of Ang-2 into the bloodstream [144]. Cellular experiments have demonstrated that Ang-2 sensitizes endothelial cells to TNF-α, promoting the modulated expression of endothelial cell adhesion in response to TNF-α [145]. Inducing podocyte-specific Ang-2 overexpression in mice results in more proteinuria and apoptosis of GECs, which is detrimental to the integrity of GECs and the filtration barrier function [80].

The balance between Ang-1 and Ang-2 is crucial in determining Tie-2 activation, which, in turn, leads to either protective or damaging effects in the interaction between podocytes and endothelial cells. These findings provide evidence for crosstalk between GECs and podocytes in the glomerulus [70]. In the latest study, an unbiased proteomic approach and integrative bioinformatics analysis identified plasma Ang-2 as a potential prognostic biomarker for DKD [146]. This finding is supported by its potential functional effects on glomerular pathogenesis in DKD through the Ang/Tie signaling pathway in endothelial cells. These results have been cross-validated in multiple cohorts [146].

The Ang/Tie ligand-receptor system also plays a role in the cellular crosstalk between MCs and GECs under HG conditions. Under HG conditions, up-regulated Ang-2 has been found to induce MC apoptosis by activating Tie-2 receptors and inhibiting miR-33-5p [81]. Additionally, in an experimental model of mesangial proliferative glomerulonephritis (MPGN), an in vitro co-culture system revealed that activated MCs promoted the proliferation of GECs. MC-derived VEGF-A stimulated the expression of Ang-2 in GECs, leading to the inhibition of Tie-2 phosphorylation and the promotion of GECs’ proliferation [147]. This suggests that there is crosstalk between MCs and GECs.

Furthermore, researchers have explored the crosstalk between proximal tubular epithelial cells (PTECs) and MCs in DKD. They found that PTEC-derived exosomal miR-92a-1-5p alters endoplasmic reticulum stress (ERS) and myofibroblast transdifferentiation in MCs in both in vivo and in vitro models [148], and this alteration contributes to the progression of DKD. These findings highlight the complex interplay between different cell types within the kidney and how it contributes to the pathogenesis of DKD.

#### Vascular endothelial growth factor (VEGF) signaling pathways

VEGF-A, a pivotal regulator of angiogenesis and vascular permeability, plays a significant role in renal function and the pathogenesis of DKD. Among various cell types, podocytes are noteworthy for their substantial VEGF-A production during fetal development. In the context of kidney physiology, VEGF is indispensable for glomerular development due to its precise control over the proliferation and survival of recruited endothelial cells, crucial for establishing and maintaining the GFB [17, 149].

In the mature glomerulus, podocytes produce VEGF-A and alter signal transduction through the VEGF-A receptors on GECs named VEGFR-1 and VEGFR-2 [150]. After fully differentiated, podocytes continue to secrete VEGF-A at low levels, sustaining endothelial fenestration and preserving endothelial function [82]. However, in the early stages of DKD, there is a significant increase in VEGF-A and VEGFR2 expression, and inhibition of the VEGF-A/VEGFR2 pathway can lead to the loss of the healthy fenestrated phenotype, further exacerbating microvascular injury in the kidney [151]. VEGF-A demonstrates dual roles, acting both as a repair-promoting and fibrosis-promoting factor, with its expression closely tied to renal fibrosis development [152]. Early renal fibrosis, triggered by inflammation and hypoxia, can stimulate heightened VEGF-A expression, thereby promoting angiogenesis, facilitating kidney self-repair, and delaying renal fibrosis [153]. However, the decrease in VEGF-A levels may be associated with glomerulosclerosis as DKD progresses [154]. These observations underscore the importance of precisely regulating VEGF-A expression to maintain normal kidney structure and function because both high and low levels of VEGF-A production can accelerate the development of DKD.

Intraglomerular interactions, influenced by the VEGF-A pathway, play a pivotal role in DKD progression. The HG condition up-regulates VEGF-A production in podocytes [83], potentially leading to endothelial cell damage. Conversely, glomerular endothelial cells can reciprocally influence podocytes through VEGF-A signaling. Elevated levels of miR-200c, found in the urine of kidney disease patients, negatively impact glomerular homeostasis by targeting VEGF-A in podocytes, resulting in glomerular damage, characterized by extensive foot process effacement and proteinuria [84]. These findings suggest a delicate balance in podocyte-endothelial cell crosstalk mediated by VEGF-A signaling in the progression of DKD. Recent research has highlighted the indispensable role of Beclin-1 in podocytes, indicating its crucial involvement in VEGF-A secretion and endothelial integrity maintenance, further emphasizing the existence of essential secretory processes underlying podocyte-endothelial crosstalk [85].

Semaphorin 3A (Sema3A), predominantly produced by mature podocytes, is a negative regulatory factor critical for podocyte differentiation and the survival of developing GECs [86]. Loss of Sema3A leads to aberrant renal vascular morphology, excessive glomerular endothelium, wide podocyte processes, and proteinuria. Paradoxically, overexpression of podocyte Sema3A during organogenesis results in glomerular hypoplasia, characterized by endothelial cell apoptosis [86]. In podocytes, endothelial cells, and collecting tubules, Sema3A transcripts localize to VEGF-A expression sites, while Sema3A receptors, neuropilins-1 (NRP1) and neuropilins-2 (NRP2), localize to VEGFR2 expression sites [155, 156]. Therefore, due to such adjacent binding sites, there exists a binding competition between VEGF-A signaling and Sema3A signaling, and it has been reported that excess Sema3A signaling interferes with VEGF-A signaling [86]. The competitive binding relationship between VEGF-A and Sema3A signaling exists [157], potentially mediated through NRP1 binding [158]. Maintaining a balance in Sema3A and VEGF-A expression in podocytes is crucial for preserving the integrity of the GFB in adult kidneys [159]. Therefore, both the Sema3A/NRP1 signaling pathway and the VEGF-A/NRP1 signaling pathway likely contribute to endothelial-podocyte crosstalk in DKD. Recent studies have unveiled the role of long non-coding RNAs T-cell factor 7 (lncRNA TCF7) in regulating Sema3A levels through direct negative regulation of miR-16-5p, shedding light on the intricate regulatory mechanisms [160]. The result shows that the loss of lncRNA TCF7 can promote the miR-16-5p level, thereby decreasing Sema3A expression to alleviate HG-induced podocyte damage [160]. Moreover, perturbations in Sema3A expression, induced by overexpression, hinder the protective effects of miR-15b-5p and exacerbate HG-mediated cell apoptosis, oxidative stress, and inflammatory responses [161].

VEGF-B governs endothelial fatty acid uptake through the transcriptional regulation of vascular fatty acid transport proteins [162]. Elevated VEGF-B levels contribute significantly to renal lipotoxicity in DKD, whereas pharmacological inhibition of VEGF-B signaling reduces renal lipid accumulation and alleviates DKD [163]. Conversely, VEGF-C demonstrates therapeutic potential in DKD. Inducing VEGF-C overexpression in podocytes has been shown to mitigate the loss of glomerular endothelial fenestrations in a model of type 1 diabetes [87]. Early evidence indicates that exogenous VEGF-C treatment can reverse the neutralizing effect of VEGF-A, while exogenous VEGF-A abolishes the ablative effect of VEGF-C in podocytes, underscoring the intricate balance of VEGF-A and VEGF-C in influencing the progression of glomerular diseases through the modulation of cellular crosstalk between glomerular endothelial cells and podocytes [164].

MCs contribute to the development of DKD through cellular interactions with podocytes and glomerular endothelial cells mediated by VEGF-related signaling pathways. MCs facilitate the differentiation of glomerular endothelial cells toward vascularization through direct cell-cell contact mediated by VEGF [165]. By generating transgenic mice with podocyte-specific VEGF overexpression, researchers have observed not only changes in endothelial cell phenotypes but also a significant decrease in many MC markers, such as α-SMA, desmin, and PDGFR-β, compared to wild-type mice, possibly due to the inhibition of PDGF-B-mediated signaling [88]. Overexpression of VEGF-A derived from podocytes has been reported to induce mesangial expansion in adult mice [89]. Furthermore, differentiated MCs are not found in the glomeruli of VEGF^loxP/loxP^ Cre^+/−^ mice, underscoring the critical role of VEGF-A production in podocytes for the survival and differentiation of MCs [90].

#### Endothelin-1 (Edn1)/Edn1 receptor type A (Ednra) signaling pathways

It is well-established that focal segmental glomerulosclerosis (FSGS) arises from podocyte-endothelial crosstalk mediated by Edn1/Ednra-dependent mitochondrial dysfunction. Edn1, secreted by podocytes, triggers endothelial cell destruction through the activation of oxidative stress [91]. Activation of TGFβR1 in podocytes rapidly induces the synthesis of pre-Edn1 and the release of Edn1 via SMAD-dependent signal transduction, both in vivo and in vitro [91] This, in turn, activates the paracrine Ednra, leading to subsequent oxidative stress within the mitochondria of GECs [91]. Inhibition of Ednra, the use of mitochondrial-targeted ROS scavengers, and endothelin antagonists have proven effective in preventing endothelial dysfunction-dependent podocyte depletion and improving albuminuria [91]. Furthermore, a study in primary podocytopathy demonstrated that the interaction between podocyte-derived Edn1 and Ednra in GECs activates mitochondrial ROS, endothelial surface layer (ESL) degradation, and remodeling pathways. This elucidates the mechanism underlying podocyte-mediated endothelial damage and dysfunction, providing insights into the loss of glomerular filtration integrity [166].

The susceptibility to DKD is closely linked to mitochondrial dysfunction, particularly within GECs. This dysfunction is associated with elevated circulating Edn1, which represents an early event in the progression of DKD [92]. Notably, selective Ednra blockade or the use of mitochondrial-targeted ROS scavengers can prevent endothelial oxidative stress and ameliorate mitochondrial ROS in GECs induced by high glucose levels, podocyte loss, albuminuria, and glomerulosclerosis [92]. In a recent study, SUMO-specific peptidase 6 (SENP6) was found to mitigate HG-induced podocyte loss and GEC dysfunction by inhibiting Edn1-mediated crosstalk between podocytes and GECs. Conversely, the supernatant from SENP6-deficient podocytes exacerbated the aforementioned GEC dysfunction [13].

In contrast to the antagonistic effects of Ednrb in GECs, it has been discovered that diabetic mice with a podocyte-specific double deletion of Ednra and Ednrb not only prevent podocyte loss but also inhibit mesangial expansion [93]. This effect may be attributed to the inhibition of β-catenin and NF-κB signaling pathways mediated by endothelin receptors. These findings underscore the significance of the pathological crosstalk between podocytes and mesangial cells in influencing the progression of DKD.

#### Endoplasmic reticulum stress (ERS) and mitochondrial oxidative stress

Given the central role of the endoplasmic reticulum in protein folding, transportation, and biogenesis, ERS can induce pathological changes resulting from cellular metabolic disorders. The analysis of cDNA microarray data from diabetic mouse glomeruli has indicated the involvement of ERS in the pathophysiology of DKD [167]. Podocytes, due to their high protein folding capacity and elevated anabolic or catabolic activity, exhibit heightened sensitivity to ERS. Elevated HG can induce ERS in podocytes, contributing to the onset and progression of DKD [168]. Although current research on ERS in GECs in the context of DKD is limited [169], ERS has been associated with cellular communication between MCs and podocytes. In an in vitro experiment, the stimulus from MCs’ supernatant cultured under HG conditions was found to inhibit the expression of ER-related degradation (ERAD)-related proteins in podocytes, such as phosphorylated IRE1α, Derlin-1, and Derlin-2. This inhibition resulted in the exacerbation of albuminuria and increased podocyte apoptosis [167]. This suggests that intraglomerular crosstalk between MCs and podocytes can impede the physiological ERAD process, leading to renal protein phosphorylation and ultimately podocyte damage in diabetes. Targeting the ERAD pathway based on this crosstalk may present a novel strategy for DKD treatment.

Mitochondrial dysfunction and oxidative stress are critical pathogenic mechanisms leading to end-organ damage in DKD. However, the understanding of these mechanisms at the cellular level remains limited. To explore the cellular mechanisms of GEC dysfunction in diabetes and its impact on adjacent podocytes, a study introduced the concept that mitochondrial stress is pivotal for GECs to release secreted factors capable of directly affecting podocytes. Following exposure to HG, GECs in mice exhibited diminished mitochondrial function, reduced endothelial function, decreased nitric oxide synthase (NOS) activity, elevated mitochondrial superoxide levels, accumulation of oxidized products (8-oxoG), disrupted mitochondrial architecture (double-strand breaks), and increased apoptotic cell death [94]. The study went on to isolate soluble factors from the cultured supernatant of stressed GECs and transfer them to co-cultured podocytes. This resulted in cell shrinkage, podocyte detachment, elevated podocyte apoptosis, increased caspase 3 levels, and cytoskeleton rearrangement. Importantly, this paracrine effect disappeared when the supernatant was derived from GECs treated with HG and a specific scavenger of mitochondrial superoxide production (mitoTEMPO) [94]. These findings support the notion that pro-apoptotic paracrine signaling factors are secreted by stressed GECs, and this secretion is necessary and sufficient to increase podocyte apoptosis, thereby highlighting the crosstalk between GEC signaling and podocytes in the context of mitochondrial oxidative stress [94].

#### Gasotransmitters-mediated signaling pathways

Over the past two decades, a growing body of literature has underscored the significant role of gasotransmitters in both biology and medicine. Coined in 2002, the term “gasotransmitter” refers to a group of small gaseous molecules, including nitric oxide (NO), hydrogen sulfide (H2S), carbon monoxide (CO), and potentially other gases [170–172]. Gasotransmitters are endogenously produced by specific enzymes and typically possess high lipid solubility, enabling them to penetrate cell membranes without the need for specific transport proteins or receptors. They exert diverse functions by targeting specific cellular and molecular targets at physiologically relevant concentrations [173].

Within the glomerulus, gasotransmitters play a pivotal role in the intricate crosstalk among MCs, podocytes, and GECs [41, 174]. Gasotransmitter-producing enzymes, including inducible nitric oxide synthase (iNOS), endothelial nitric oxide synthase (eNOS), heme oxygenase (HO-1), cystathionine-γ-lyase (CSE), and cystathionine beta synthase (CBS), have been identified in various glomerular cell types, such as GECs, podocytes, and MCs. However, not all of these enzymes are expressed in each cell type. Instead, a finely tuned crosstalk exists among these three types of glomerular cells during the synthesis and action of gasotransmitters, contributing to the maintenance of glomerular homeostasis [175, 176].

NO, a potent endothelial-derived vasodilator, is believed to play a crucial role in regulating renal blood flow, glomerular filtration rate, and mesangial matrix accumulation. Studies have shown the presence of all three NOS isoforms in the glomerulus of both human and rodent species (available online: https://susztaklab.com). However, limited research has focused on the individual expression of NOS subtypes in specific glomerular cell types. Among these, eNOS is expressed in GECs, producing low levels of NO and is considered a protective mechanism against glomerular diseases. Under physiological conditions, it serves as the primary producer of NO in the glomerulus [177]. Diabetic eNOS-null (STZ-eNOS^−/−^) mice exhibited a higher degree of apoptosis, oxidative stress, and proliferation compared to STZ-WT GECs. This suggests that eNOS plays a significant role in the dysregulation of angiogenesis and epigenetic regulation in DKD [120]. Additionally, STZ eNOS^−/−^ mice displayed biphasic changes in the number of GECs, characterized by an initial increase followed by a decrease over time [120]. This pattern aligns with the early compensatory angiogenesis process, succeeded by an increase in cell apoptosis, ultimately leading to more GEC death during the progression of DKD [120].

Potential candidate genes, such as leucine-rich alpha 2 glycoprotein (LRG1) and G protein-coupled receptor-56 (GPR56), enhancing angiogenesis induced by diabetes, have been identified [120], and GPR56 has been found to promote DKD through regulating eNOS mediated by coupling of Gα12/13-RhoA pathway activation and Gαi-mediated cAMP/PKA pathway inhibition [178]. Moreover, the expression of eNOS has been observed to increase under HG conditions. One study demonstrated that HG increased eNOS protein expression but ultimately reduced NO release, seemingly due to excessive superoxide production and L-arginine deficiency. This finding elucidates the molecular basis of how elevated glucose levels lead to an imbalance between NO and superoxide production, resulting in impaired endothelial function [179].

An active constitutive NOS isoform has been identified in MCs, and under HG conditions, enhanced eNOS mRNA and protein expression led to increased NO production in MCs, potentially contributing to hyperfiltration in DKD [180]. In contractile MCs, NO may induce relaxation and act as a pro-inflammatory mediator, rapidly upregulated in response to inflammatory cytokines. Research has demonstrated that interleukin-1β (IL-1β) induces the expression and activation of NOS2 in MCs [181]. Furthermore, in diabetic eNOS-deficient mice, there is a tendency for mesangial lysis and late mesangial dilatation, likely resulting in nodular or Kimmelstiel-Wilson-like lesions, indicative of DKD development [47, 97]. Evidence has shown that GECs can interact with both MCs and podocytes through eNOS-related mechanisms. A co-culture system of GECs and bovine MCs demonstrated that Ca++ mobilizing agonists, such as bradykinin, induced cGMP formation in MCs in a NO-dependent manner. This effect, however, disappeared when MCs were cultured in the absence of GECs [95]. These findings suggest that GEC-derived NO affects MCs, potentially influencing the regulation of intraglomerular capillary flow through this finely tuned cellular crosstalk.

Podocytes have been shown to produce NO and presumably express NOS1 under normal physiological conditions [182, 183]. PCR-based studies have also suggested the expression of NOS2 and NOS3 isoforms in podocytes [184]. Reduced NO production in podocytes may contribute to disease progression [96]. Notably, podocytes respond to NO derived from GECs in a paracrine manner, with endothelium-derived NO serving to maintain podocyte structure and function [98].

#### Hypoxia inducible factor (HIF)-related signaling pathways

Hypoxia-induced dysregulation of intraglomerular cell interactions is implicated in the development of DKD. Hypoxia can lead to severe renal tubulointerstitial injury and loss of peritubular capillaries. However, the extent of hypoxia-induced glomerular capillary damage is much milder compared to tubulointerstitial damage. The reasons for this difference remain unclear. A study has uncovered that this potential protective effect is attributed to the crosstalk between podocytes and GECs [99].

Hypoxia can promote the stabilization and activation of HIF-1α by increasing SENP1 expression in podocytes, thereby sustaining the survival of GECs and promoting angiogenesis through the VEGF/VEGFR2 signaling pathway to counteract hypoxia. Furthermore, blocking deSUMOylation induced by SENP1 shRNA effectively inhibited the activation of HIF-1α signaling, ultimately negating this protective effect [99]. Consequently, deSUMOylation plays a crucial role in HIF-1α signaling activation in podocytes, and SENP1 emerges as a potentially novel therapeutic target for the treatment of hypoxic renal disorders [99].

HIF-2α represents an endothelial subtype of HIF known as endothelial Per-ARNT-Sim domain protein 1 (EPAS1). Endothelial-specific deficiency of EPAS1 can lead to the loss of fenestration in GECs and an increase in endothelial swelling. Dysfunction of GECs is associated with the disappearance of podocyte processes, leading to the aggravation of proteinuria and glomerulosclerosis [185].

Moreover, in the presence of hypertension and EPAS1, no podocyte lesions were observed, indicating that endothelial-specific EPAS1 gene deletion exacerbates proteinuria and results in severe podocyte damage [185]. Currently, there is still ongoing debate regarding the impact of HIF-α on inflammation, oxidative stress, and fibrosis in DKD, as well as the intricacies of glomerular cell-to-cell crosstalk, all of which necessitate further in-depth exploration [186].

#### Krϋppel-like factors (KLFs) signaling pathways

The Krüppel-like factor (KLF) protein is a pivotal regulatory factor within the physiological system, encompassing the cardiovascular, hematologic, respiratory, digestive, and immune systems. KLFs exert control over critical physiological processes in the kidney, such as maintaining the normal functioning of the GFB [187]. Furthermore, they are also implicated in pathological processes, including tubulointerstitial inflammation and renal fibrosis [188]. KLF2 primarily manifests in endothelial cells, where it shields them from cellular stress [189]. Wall shear stress, the hydrodynamic frictional force generated by blood flow, is predominantly perceived by the endothelial glycocalyx, resulting in the regulation of the expression of KLF2 through signal transduction. This regulation is pivotal for the preservation of GEC function [190]. In early DKD, KLF2 expression is induced by glomerular hyperfiltration, and its transcription is suppressed by HG levels but enhanced by insulin [100]. A study has shown that endothelial cell-specific KLF2 heterozygous knockout mice (DKD mice model by STZ injection) developed more albuminuria than the wild type mice; in addition, the expression of podocyte-specific genes(nephrin, synaptopodin, podocin, and podocalyxin) were also down-regulated, together with more podocyte damage, suggesting a potential crosstalk between GECs and podocytes [100]. The result also showed elevated expression of angiogenesis markers such as VEGF-A, Flk1, and Ang-2, along with reduced expression of Flt1, Tie2, and Ang-1 [100]. This likely indicates intricate interactions among these angiogenesis-related signaling pathways. Another study also similarly found that GEC-specific KLF2 heterozygous knockout mice exhibited a significant reduction in the number of podocytes and the expression of podocyte markers, further indicating the existence of potential crosstalk between GECs and podocytes [101]. However, the precise mechanism governing this crosstalk requires further investigation.

The crosstalk between GECs and podocytes can also be influenced by laminar shear stress (LSS). GECs can release mediators dependent on chronic LSS, which in turn affect podocyte signaling and behaviors. A study has revealed that one of the mechanisms involved is the increased expression of KLF2 and downstream molecules mediated by ERK5. This leads to improvements in anticoagulant and anti-inflammatory phenotypes, directly impacting podocyte function in co-culture [102].

Presently, research on the role of KLF15 in renal biology primarily centers on podocyte injury, mesangial pathology, and renal fibrosis [191]. While podocyte-specific KLF15-knockout mice do not exhibit significant proteinuria or glomerulosclerosis under undisturbed conditions, their susceptibility to podocyte damage significantly increases in mouse models induced by lipopolysaccharide (LPS) or doxorubicin (ADR). This indicates that KLF15 may emerge as a potential regulatory factor for podocyte differentiation and protection against damage [192]. Notably, KLF15 is highly expressed in GECs and MCs [193], and its overexpression inhibits the cell cycle of MCs and eliminates MC proliferation mediated by SUMO1 [194]. However, direct evidence demonstrating the interaction between KLF15 and the three types of glomerular cells mentioned above is currently lacking. Further studies are needed to elucidate this aspect.

#### Retinoic acid receptor response protein 1(RARRES1) signaling pathways

Retinoic acid (RA) receptor response protein 1 (RARRES1) has been identified as a novel protein specifically expressed in podocytes, a finding verified through single-cell RNA sequencing [195]. RA exerts pleiotropic effects on cellular behavior, including the induction of cell differentiation while inhibiting proliferation and inflammation [196, 197].

In the context of kidney diseases, RA can promote apoptosis of podocytes and their loss by suppressing the expression of RARRES1, which has recently been described as a risk factor for the progression of glomerular diseases. While most current investigations into RARRES1 primarily center on podocytes [198], it’s important to note that the release of RARRES1 fragments into the extracellular space may also impact other cells within the kidney, especially adjacent MCs, parietal epithelial cells, and capillary endothelial cells in the glomerulus. Additionally, RARRES1 fragments may potentially leak into the renal tubular lumen, influencing renal tubular cells and serving as messengers in intercellular crosstalk during the development of glomerulopathy and chronic kidney disease [199].

Molecular profiling of patients with diabetic kidney disease (DKD) has indicated that RARRES1 is produced in glomerular endothelial cells (GECs) but is not detected in diabetic patients without DKD [103]. This suggests that RARRES1 derived from GECs may play a significant role in DKD. Current research has found that RARRES1 overexpression in GECs induces podocyte injury by activating the NK-κB signaling pathway through the receptor tyrosine kinase Axl [103]. One plausible explanation for this is that the soluble form of RARRES1 produced by podocytes is taken up by GECs. However, it’s worth noting that RARRES1 mRNA has also been detected in GECs [103]. Consequently, it remains uncertain whether this pathway is activated by RARRES1 in GECs, podocytes, or both. In the future, further studies can be designed to confirm the following possibilities: firstly, RARRES1 induces changes in GECs through autocrine effects, subsequently causing podocyte damage through crosstalk, which may involve the release of cytokines; secondly, soluble RARRES1 released from endothelial cells acts on podocytes, inducing damage through paracrine effects [200].

#### Platelet-derived growth factor B (PDGF-B)/platelet-derived growth factor receptor β (PDGFR-β) signaling pathways

PDGF is a major mitogen for fibroblasts as well as smooth muscle cells, playing an important role in embryonic development, wound healing, and the vascular system [201]. In glomeruli, PDGF and PDGFR mRNAs are mainly expressed in glomerular resident cells [106]. PDGF-B localizes to the GECs, and PDGFR-β localizes to the MCs [104]. In a normal condition, the PDGF-B derived from GECs can recruit MCs into developing glomeruli and promote the formation of capillary rings [105]. In diabetes, the dysfunction of GECs is one of the earliest events existing in the normal albuminuria stage of diabetes, which may promote DKD by releasing paracrine signaling mediators to cause MCs’ damage [107]. A study has found that the expression of both PDGF-B and PDGFR-β were up-regulated in the histologically early stage of DKD [106]. The HG condition can increase PDGF-B/PDGFR-β expression and lead to MCs’ proliferation and mesangial expansion [108], which suggests the potential communication between GECs and MCs through PDGF-B/PDGFR-β signaling. Besides, as a key factor of DKD, hypoxia can stimulate the increase of endothelial PDGF-B mRNA and enhance the specific binding capacity between PDGF-B and PDGFR-β, thus regulating the PDGF-B paracrine interactions between GECs and MCs, which causes mesangial expansion [109].

#### Slit–roundabout receptor (Robo) signaling pathways

Studies have found that several Slit–roundabout receptor (Robo) pathways may participate in the interaction between MCs and GECs. Researchers found induction of a secreted factor in MCs called Slit3 whose receptor was detected in GECs and podocytes in IgA nephropathy (IgAN) [202], indicating the potential crosstalk among these cells. In human DKD, a single-cell transcriptomics study also detected the induction of Slit3 in MCs [203]. Slit2 is a secreted polypeptide that binds to transmembrane Robo receptors [110] and Slit2/Robo signaling regulates axon guidance, ureteric bud branching, and angiogenesis during kidney development as well as glomerular filtration in adult kidneys [111].

Researchers have found that the expression of Slit2 and VEGF in human renal MCs significantly increased after treatment with HG. Moreover, Slit2/Robo1 signaling is activated in GECs treated with HG-MCs, promoting the angiogenic activity of GECs via the Robo1/PI3K/Akt/VEGF pathway. Additionally, blockade of Slit2/Robo1 signaling inhibited HG-MCs-induced GECs proliferation, migration, and tube formation [112]. Therefore, these results suggest that Slit2/Robo1 signaling participates in HG-MCs-induced GEC angiogenesis, and Robo1 may be a potential therapeutic target in abnormal angiogenesis in DKD. Furthermore, after transfection with Slit2 siRNA to remove the effect of Slit2 derived from MCs, it was demonstrated that there is also an autocrine activation of Slit2/Robo1 signaling in GECs, contributing to GEC angiogenesis through the PI3K/Akt and HIF-1α/VEGF signaling pathways [112].

#### Bone morphogenetic protein 4 (BMP4)-Smad1 signaling pathways

Previous studies have established a close relationship between Smad1 and mesangial expansion in DKD [113, 114]. In mice with DKD and podocyte-specific overexpression of bone morphogenetic protein 4 (BMP4), not only podocyte loss but also mesangial expansion occurred [115]. This can be attributed to the activation of Smad1 in MCs induced by BMP4 expression in podocytes.

## Application of new technologies in studying cell interaction in DKD

Currently, methods for studying cell-cell interactions are continuously evolving. The following section introduces several commonly employed techniques for investigating cell-cell interactions, which include single-cell RNA-sequencing, spatial transcriptomics, and biomimetic in vitro systems, such as kidney organoids and kidney on-a-chip models. It also provides a summary of their advantages and limitations (Table 2).

**Table 2:** Application, advantages and limitations of technologies in studying glomerular cell crosstalk in DKD

Technology		Application	Related research	Advantages/Limitations	Reference
Single-cell RNA-sequencing		CellPhoneDB: a new repository of ligands, receptors and their interactions, which is more accurate to represent heterogeneous complexes compared to others.	It has been used to analyze scRNA-seq data on a public dataset to identify the cell-cell crosstalk networks in DKD;	• Advantages: Not mentioned. • Limitations: 1) The difficulty of isolating glomerular cells from core needle biopsy specimens; 2) No effective standardized pipelines are available at present; 3) Unspecific available cell or unrevealed cell markers; 4) Requiring appropriate analytical and statistical methods, depending on the choice of computing tools and databases. 5) Relying on the affymetrix microarray dataset to infer the pathogenesis of diabetes nephropathy, and the human transcriptome data is limited.	[204, 205]
		CellChat: a tool that can quantitatively infer and analyze cellular interaction networks from scRNA-seq data and performs well in predicting stronger interactions, which helps to narrow the range of interactions for further experimental validation.	It has been applied to discover the dysfunctional signaling and metabolic pathways in the thin endometrium, which provides insights into the mechanisms and treatment strategies of atrophic endometrium;		[206, 207]
		ICELLNET: a global, versatile, biologically validated, and easy-to-use framework to analyze cell crosstalk from individual or multiple cell-based transcriptomic profiles.	1) It has been applied to three datasets generated based on RNA-seq, scRNA-seq, and microarray; 2) It has revealed autocrine IL-10 control of human dendritic cell communication with up to 12 cell types;		[208]
		SMARTseq2 technology: a technology used for scRNA-seq analysis of glomerulus-associated cells.	1) It showed the proximal tubular cells were strongly affected at an early stage of IgA nephropathy and a potential glomerular-tubular cell-cell crosstalk pathway was identified; 2) Researchers also explored the early human DKD via single-cell transcriptomic landscape and showed the altered signaling networks in the diabetic glomerulus among GECs, podocytes and MCs;		[122, 202]
Spatial transcriptomics		Spatial transcriptomics: an emerging new technology that provides quantitative gene expression data and visualization of mRNA distribution in tissue slices.	A study explored the cell-cell interactions and signaling networks in the cell subsets using ligand-receptor analysis and visualized potential interactions in different kidney cell types;	• Advantages: It can quantify the mRNA levels of thousands of genes in the entire tissue slice and the mRNA levels in the tissue slice, allowing for the identification of direct connections between histological findings and gene expression. • Limitations: Not mentioned.	[136, 209]
Biomimetic systems in vitro	Kidney organoid	Kidney organoid: a self-organizing, three-dimensional aggregate of cells, derived from embryonic stem cells and induced pluripotent stem cells (iPSCs).	A study developed the first diabetic kidney organoid model to explore the potential mechanisms underlying the increased disease severity in patients with COVID-19 and diabetes;	• Advantages: It may be the first time to allow in vitro research on Proteinuria, to realize mechanism and preclinical research. • Limitations: Different protocols and different measurements need to be standardized.	[210–213]
	Microfluidic bioreactor	The microfluidic bioreactors: an emerging technology allowing continuous infusion of culture medium and secretion factors during the renal patterning process.	It has been used to coculture endothelial cells(ECs) with human kidney proximal tubule epithelial cells(HPTECs), which showed an increased upregulation of kidney-specific genes and suggested a potential bidirectional paracrine signaling;	• Advantages: It shows promise in enhancing both renal organoid differentiation and cell type and also allows for the simultaneous generation of paracrine signals between two separated cell populations through shared media, allowing for the exchange of soluble factors and transient signals. • Limitations: Not mentioned.	[214]
	Kidney on-a-chip	Glomerulus on a chip: a new in vitro organ model developed in the field of organ chip research based on microfluidic device technology. Podocytes and endothelial cells are co-cultured in a microfluidic device in the glomerulus on the chip with physical stimuli that simulate physiological environments to enhance cell function and construct functional filtration barriers.	1) A new customized chip-based glomerular co-culture model has been developed and demonstrated that the co-culture affected the morphology and transcriptional phenotype of glomerular endothelial cells and podocytes; 2) A study used a glomerulus-on-a-chip microdevice that reconstitutes organ-level kidney functions to create a human disease model of early-stage DKD on a chip.	• Advantages: The co-culture improves barrier function as a relevant functional readout for clinical translation, and this has been used to investigate specific glomerular paracrine pathways and unravel the role of glomerular crosstalk in glomerular path/physiology. • Limitations: Not mentioned.	[215, 216]

### Single-cell RNA-sequencing

Single-cell RNA sequencing (scRNA-seq) stands as a powerful tool that has been harnessed to offer unprecedented insights into the cellular transcriptome, including the elucidation of cell-to-cell communication in diseases like DKD. ScRNA-seq has enabled researchers to uncover novel complex cellular interactions within DKD and identify new cellular subpopulations within the kidney. These findings shed light on key regulatory factors and potential therapeutic targets for DKD. In recent years, researchers have identified several ligand-receptor pairs involved in the crosstalk among podocytes, GECs, and MCs in control and diabetic mice. While some of these pairs are well-established (e.g., podocyte VEGFA-endothelial Flt1 and Kdr), others are less characterized in glomerular homeostasis (e.g., mesangial Epha3-endothelial Efna1) [119]. Future studies are required to unravel their interactions and unveil their roles in diabetic glomerular disease. ScRNA-seq analysis has facilitated the confirmation of specific markers and genes expressed in glomerular cells, leading to the identification of several new potential markers for glomerular cells in DKD [136].

CellPhoneDB, a novel repository of ligands, receptors, and their interactions, offers a more accurate representation of heterogeneous complexes compared to other repositories. It has been employed to analyze scRNA-seq data from a public dataset to discern the cell-cell crosstalk networks in DKD [204]. Researchers have provided a step-by-step guide for implementing the CellPhoneDB protocol, which allows for the inference of cellular crosstalk networks from scRNA-seq data [205]. Additionally, CellPhoneDB v.2.0 has enhanced functionalities, including the introduction of new interacting molecules and a reduction in the time and resources needed to query large datasets, among other improvements [205].

CellChat has the capability to quantitatively infer and analyze cellular communication networks via scRNA-seq analysis, facilitating a deeper understanding of cellular interactions among various cell types [206]. It excels at predicting stronger interactions, helping to focus subsequent experimental validation efforts [206]. This tool has been applied to uncover dysfunctional signaling and metabolic pathways in the thin endometrium, providing valuable insights into the mechanisms and treatment strategies for atrophic endometrium [207]. Cell-to-cell communication can also be inferred from ligand-receptor expression patterns based on the transcriptome.

ICELLNET represents a biologically validated, user-friendly, versatile, and globally applicable framework for profiling cellular communication based on single or multiple cell-based transcriptome profiles. It has been successfully applied to analyze three datasets generated by RNA-seq, scRNA-seq, and microarrays. These analyses have unveiled the autocrine control of IL-10 in human dendritic cell communication, encompassing interactions with up to 12 different cell types [208].

For the scRNA-seq analysis of glomerulus-associated cells, SMARTseq2 technology was employed, yielding results that unveiled a potential glomerular-tubular cell-to-cell crosstalk pathway identified at an early stage of IgA nephropathy. The functionality of these key crosstalk pathways was subsequently validated using cell culture models [202]. Furthermore, researchers have delved into the altered signaling networks within the diabetic glomerulus, involving GECs, podocytes, and MCs, through a single-cell transcriptomic landscape analysis conducted during the early stages of human DKD [122].

While technology has made significant advancements, researchers still encounter several challenges. Firstly, the isolation of glomerular cells from core needle biopsy specimens for single-cell transcriptomics remains a challenging task [217]. The kidney possesses a relatively dense stroma, and kidney cells are susceptible to loss under abnormal conditions. Therefore, there is a need to optimize cell separation protocols to enhance efficiency while balancing the effects of cell separation and cell viability [217, 218]. Presently, there are substantial variations in the number of renal cells obtained and in gene expression across different renal single-cell RNA sequencing (scRNA-seq) studies. These differences are largely attributed to variations in dissociation protocols, but effective standardized pipelines are currently lacking [217, 218]. The identification of renal cell types in scRNA-seq studies primarily relies on available cell markers, which may lack specificity or even remain undiscovered [218]. Additionally, the vast amount of complex data generated by scRNA-seq demands appropriate analytical and statistical methods. The interpretation of raw data hinges on the selection of computational tools and databases [218]. Most importantly, the results obtained from scRNA-seq require validation through subsequent experimental tests211. It is worth noting that single-nuclei RNA sequencing (snRNA-seq) may offer advantages over scRNA-seq in the isolation of glomerular cells, although its efficacy with core needle biopsy specimens remains to be established [217].

### Spatial transcriptomics

Spatial transcriptomics (ST) is an emerging technology that provides quantitative gene expression data and visualizes mRNA distribution within tissue slices. It can quantify mRNA levels of thousands of genes across the entire tissue slice and pinpoint mRNA levels within the tissue slice. This enables the identification of direct connections between histological observations and gene expression [209]. A recent study investigated cell-cell interactions and signaling networks in cell subsets using ligand-receptor analysis. It visualized potential interactions among different kidney cell types, revealing stronger interactions between fibroblasts from individuals with DKD and various cell types, such as proximal tubule cells, podocytes, and endothelial cells, when compared to nondiabetic controls and individuals with DM [136]. These interactions were primarily related to chemokines and were predominantly found in fibroblasts, highlighting the close association between renal fibrosis and the immune response in the context of DKD [136].

### Biomimetic systems in vitro

#### Kidney organoid

Kidney organoids represent self-organizing, three-dimensional cell aggregates derived from embryonic stem cells and induced pluripotent stem cells (iPSCs). They closely mimic the internal environment and hold promise for organ regeneration, disease modeling, and drug screening [210]. Moreover, the development of kidney organoids and microfluidic systems has opened up the possibility of in vitro studies of proteinuric diseases for the first time, facilitating mechanistic and preclinical investigations [211].

A groundbreaking study has introduced the first DKD organoid model, employed to explore potential mechanisms underlying increased disease severity in patients with both COVID-19 and diabetes. This model achieved functional vascularization through a combination of vascular organoids and a microfluidic device platform, revealing crucial pathological features of the kidneys [212].

Nevertheless, a primary challenge in the field of kidney organoids lies in the discrepancies not only between different protocols but also between measurements. To establish a standardized framework, the American Society for Cell Biology has formulated guidelines for organoid research, aiming to promote transparent and reproducible research outcomes [213]. Microfluidic bioreactors, therefore, play a crucial role in standardizing kidney organoid generation and minimizing disparities between studies.

#### Microfluidic bioreactor

Microfluidic bioreactors represent an emerging technology that enables the continuous infusion of culture medium and secretion factors during the renal patterning process, showing great potential in enhancing renal organoid differentiation and cell type specification. Traditional in vitro disease models predominantly consist of cell models, primarily cultivated in two-dimensional (2D) static cultures. While cells cultured in this manner can retain some biological functions, they lack essential in vivo microenvironment factors such as multicellular interactions, ECM, and physiological and chemical stimulation. This limits their ability to simulate the physiological and pathological states of human tissue organs within a static cell culture environment. When investigating glomerular crosstalk, co-culturing two or more cell types adds complexity compared to single-culture models. Open microfluidic systems enable the simultaneous generation of paracrine signals between separated cell populations through shared media, facilitating the exchange of soluble factors and transient signals. For instance, microfluidic bioreactors have been employed to coculture endothelial cells with human kidney proximal tubule epithelial cells, leading to an increased upregulation of kidney-specific genes and suggesting the potential for bidirectional paracrine signaling [214].

#### Kidney on-a-chip

The kidney glomerulus on-a-chip is a relatively new in vitro organ model that was first introduced in 2016, and it has seen significant advancements in the field of organ chip research. In this microfluidic device, podocyte and endothelial cells can be co-cultured, creating an environment that enhances cell function and establishes a functional filtration barrier, all within a physiologically simulated setting. This innovative model holds promise for future research endeavors aimed at investigating specific paraglomerular secretion pathways and unraveling the intricate roles of cellular crosstalk in glomerular pathology and physiology.

The importance of crosstalk between GECs and podocytes in maintaining the integrity of the GFB is increasingly evident. However, due to a lack of suitable experimental models, there have been limited in vitro studies directly examining the impact of this crosstalk on the GFB [215]. In vivo, GEC-podocyte communication plays a crucial role in regulating the integrity and selective permeability of the GFB. Under HG conditions, podocytes can detach from the GBM and exhibit increased motility. A study employed a glomerulus-on-a-chip microdevice to recreate early-stage DKD in a human disease model. This microdevice effectively reproduced the damaged GFB observed in human DKD, showcasing increased protein permeability, ROS production, and podocyte detachment from endothelial cells [219]. These results support the notion that disruptions in cell-cell interactions within the glomerulus may lead to GFB deterioration and increased permeability, resulting in proteinuria. Additionally, the glomerular chip microdevice holds the potential to advance drug therapy development for GFB. Researchers have also designed a customized glomerulus co-culture model using soft lithography to create a chip-based glomerulus model. This approach allowed for the customization of the microfluidic device’s design, facilitating the exploration of the impact of co-culturing on the phenotypes of GECs and podocytes [215]. Bulk RNA sequencing experiments revealed profound alterations in biological pathways in both cell types due to co-culture. Interestingly, the disappearance of podocyte processes, once considered the initial step in glomerular disease pathogenesis, may also result from endothelial origin [215]. Another study developed a microfluidic model that replicates glomerular filtration physiology through adjustable GBM deposition and 3D co-culture of podocytes and GECs [216]. Precise control over GBM thickness successfully reproduced the biphasic response of the GFB. Notably, microscale proximity between GECs and podocytes promoted dynamic crosstalk essential for maintaining GFB integrity and function [216]. For example, the addition of GBM and podocytes synergistically induced an increase in GEC tight junctions. Confocal and transmission electron microscopy imaging further revealed the ultrastructure of GECs-GBM-podocytes’ foot processes in contact, enhancing our understanding of crosstalk between GECs and podocytes [216].

## New promising mediators for cell crosstalk in DKD

The vast majority of cells possess migratory capabilities and can release small vesicles to facilitate communication with other cells. A recent study has unveiled a novel mechanism known as “migracytosis” that mediates the release of intracellular substances. This process is orchestrated by a newfound organelle featuring vesicular structures referred to as “migrasomes” [220]. Migrasomes are produced at the intersections or distal tips of long tubular structures known as “retraction fibers,” which extend behind the cell body. During the migracytosis process, migrasomes connect to the cell body via retraction fibers. Subsequently, upon the breaking of retraction fibers, the small vesicles contained within migrasomes are released either into the extracellular matrix or are directly ingested by adjacent cells, thereby facilitating communication between cells.

Presently, scholars have begun to focus on understanding the role of migrasomes in cell interactions within various disease mechanisms. Researchers have initiated investigations into how migrasomes may contribute to DKD. Distinct from exosomes, migrasomes released by podocytes exhibit different content and mechanisms of release. It has been substantiated that podocytes can release an increasing number of “injury-related” migrasomes during migration under HG conditions. Importantly, this release occurs earlier than the onset of proteinuria, implying that migrasomes may serve as more sensitive and reliable indicators of podocyte stress or damage [221]. Nevertheless, their involvement in the cell-to-cell interaction processes of DKD remains unclear, warranting further research efforts.

## Discussion

Studying the mechanisms of intraglomerular cell interactions is essential for a better understanding of the pathogenesis of DKD and for the early intervention in its treatment. As detailed earlier, numerous studies have reported the characteristics and significant pathogenic effects of glomerular cell crosstalk in DKD. As previously noted by other researchers, MCs also play a crucial role in maintaining glomerular homeostasis [16]. Consequently, we emphasize the role of MCs in our focus on cellular crosstalk in DKD, aiming to enhance our current comprehensive understanding of their interaction mechanisms. However, the present work lacks enough combined analysis of the cellular crosstalk among these three glomerular cell types (podocytes, GECs, and MCs) in DKD. Furthermore, there is limited information available regarding the role of intraglomerular interactions at different stages of DKD progression.

In this review, we have summarized the different signal pathways involved in cell-to-cell crosstalk among glomerular cells in DKD. Many signal pathways appear to be involved in interactions across different cell types. For example, podocytes influence GECs and MCs through pathways like VEGF signaling and Edn1 signaling. Similarly, GECs influence podocytes and MCs through pathways like TGF-β signaling, and MCs influence GECs and podocytes also via TGF-β signaling. Additionally, we can observe that TGF-β signaling and Ang/Tie signaling pathways are shared in the interaction between GECs and MCs. Therefore, it is reasonable to postulate that the mentioned pathways may hold more promising potential for targeted therapies compared to other signaling pathways.

Regarding the types of crosstalk mediators, besides the well-documented protein-based molecules, miRNAs released by exosomes also play a role in regulating cell-to-cell communication in DKD. Examples include miR-16-5p, miR-200c, and miR-33-5p, among others. Another emerging mediator for cell interaction is migrasomes, which are expected to become a prominent topic in the study of cell interaction in DKD in recent years.

Regarding pathological pathways, in addition to the well-established classical interaction pathways such as PDGF-B/PDGFR-β signaling, VEGF signaling, Ang/Tie signaling, and Edn1 signaling, some recently studied pathways like KLFs signaling, HIF-1α signaling, and Slit2/Robo1 signaling are also found to participate in crosstalk. Furthermore, the location of these pathological processes varies; for instance, KLFs signaling occurs in the nucleus, while some specific pathological processes take place in other organelles, such as endoplasmic reticulum stress and mitochondrial oxidative stress. Additionally, a study has also summarized the interaction between tubular epithelial cells (TECs) and GECs in DKD, suggesting that TECs may play a significant role in cellular interaction during the development of DKD [14], providing another avenue for potential research.

Moving from diabetes to DKD, the occurrence of proteinuria has always been a major concern. However, it remains unclear which cells are affected first based on the interaction mechanism among the three types of glomerular cells. Consequently, determining the optimal timing and approach for early intervention remains elusive. Studying the mechanisms of cell interaction is crucial for understanding the specific pathological processes underlying DKD, which, in turn, aids in developing drugs for early intervention to reduce the incidence of proteinuria.

Currently, our understanding of glomerular paracrine signaling communication has greatly benefited from the advancement of modern genetic tools, including single-cell RNA-sequencing, spatial transcriptomics, and in vitro biomimetic systems. Nevertheless, these current technologies still have limitations and require further advancement. While various sequencing techniques have been employed to integrate gene enrichment, pathway analysis, tissue-specific expression analysis, and hub gene identification in diabetic nephropathy, there are notable limitations. Most sequencing technologies, such as scRNA-seq, primarily rely on existing Affymetrix microarray datasets to infer the pathogenesis of diabetic nephropathy. Moreover, human transcriptome data itself is limited, lacking extensive clinical validation of candidate genes identified and drug perturbations. Therefore, further research in this field is necessary to open up new avenues for exploring biomarkers and the pathology of DKD.

## Conclusion

In conclusion, we have summarized the evidence indicating that interactions among glomerular cells play a crucial role in accelerating the development of DKD. The pursuit of drug development for treating DKD by targeting the pathways and targets involved in these interactions has always held promise. However, the cell-to-cell crosstalk in DKD is highly intricate, and there remain many potential channels and mediators in cell crosstalk that remain unclear, such as migrasomes. Efforts should be dedicated to unraveling these complex processes and relationships, and further exploration in this area is imperative.

## Data Availability

Permit unrestricted non-commercial use, distribution, and reproduction in any medium, provided the original work is properly cited. This article is licensed under a Creative Commons Attribution 4.0 International License, which permits use, sharing, adaptation, distribution and reproduction in any medium or format, as long as you give appropriate credit to the original author(s) and the source, provide a link to the Creative Commons licence, and indicate if changes were made. The images or other third party material in this article are included in the article's Creative Commons licence, unless indicated otherwise in a credit line to the material. If material is not included in the article's Creative Commons licence and your intended use is not permitted by statutory regulation or exceeds the permitted use, you will need to obtain permission directly from the copyright holder. To view a copy of this licence, visit http://creativecommons.org/licenses/by/4.0/. The Creative Commons Public Domain Dedication waiver (http://creativecommons.org/publicdomain/zero/1.0/) applies to the data made available in this article, unless otherwise stated in a credit line to the data.
